# Multi-Fault Diagnosis of Rolling Bearings via Adaptive Projection Intrinsically Transformed Multivariate Empirical Mode Decomposition and High Order Singular Value Decomposition

**DOI:** 10.3390/s18041210

**Published:** 2018-04-16

**Authors:** Rui Yuan, Yong Lv, Gangbing Song

**Affiliations:** 1 Key Laboratory of Metallurgical Equipment and Control Technology, Wuhan University of Science and Technology, Ministry of Education, Wuhan 430081, China; 201403703005@wust.edu.cn; 2 Hubei Key Laboratory of Mechanical Transmission and Manufacturing Engineering, Wuhan University of Science and Technology, Wuhan 430081, China; 3 Smart Material and Structure Laboratory, Department of Mechanical Engineering, University of Houston, Houston, TX 77204, USA

**Keywords:** multi-fault diagnosis, power imbalance, filter bank property, APIT-MEMD, HOSVD

## Abstract

Rolling bearings are important components in rotary machinery systems. In the field of multi-fault diagnosis of rolling bearings, the vibration signal collected from single channels tends to miss some fault characteristic information. Using multiple sensors to collect signals at different locations on the machine to obtain multivariate signal can remedy this problem. The adverse effect of a power imbalance between the various channels is inevitable, and unfavorable for multivariate signal processing. As a useful, multivariate signal processing method, Adaptive-projection has intrinsically transformed multivariate empirical mode decomposition (APIT-MEMD), and exhibits better performance than MEMD by adopting adaptive projection strategy in order to alleviate power imbalances. The filter bank properties of APIT-MEMD are also adopted to enable more accurate and stable intrinsic mode functions (IMFs), and to ease mode mixing problems in multi-fault frequency extractions. By aligning IMF sets into a third order tensor, high order singular value decomposition (HOSVD) can be employed to estimate the fault number. The fault correlation factor (FCF) analysis is used to conduct correlation analysis, in order to determine effective IMFs; the characteristic frequencies of multi-faults can then be extracted. Numerical simulations and the application of multi-fault situation can demonstrate that the proposed method is promising in multi-fault diagnoses of multivariate rolling bearing signal.

## 1. Introduction

Being crucial parts in rotating machinery and equipment, rolling bearings are of great importance. Due to their complex structure and tough working condition in industrial field, rolling bearings are prone to fail [1,2,3]. Due to the limited characteristic information of the vibration signal collected by one sensor, the vibration signal collected from a single channel tends to miss some fault characteristic information, resulting in erroneous diagnoses [4,5]. It’s meaningful to use multiple sensors to collect multivariate vibration signals from different locations, in order to extract more accurate characteristic frequencies of fault rolling bearings [6,7,8]. Especially when multiple faults occur in different rolling bearings, one sensor usually cannot collect all the fault characteristic information for subsequent fault diagnosis [9]. Hence multivariate signal processing methods are put forward to deal with multivariate signals simultaneously. 

Empirical mode decomposition (EMD) was firstly proposed to analyze nonlinear and non-stationary data adaptively, and it can decompose a time series into several approximative stationary time series, namely intrinsic mode functions (IMFs) [10]. Compared to traditional signal processing methods in the field of mechanical fault diagnosis, such as short time Fourier transform (STFT) [11], wavelet transform (WT) [12], and independent component analysis (ICA) [13], EMD has no basis function requirement. Nonetheless, EMD still has problems, such as modal aliasing and end effect. Aiming at mode aliasing problem, ensemble empirical mode decomposition (EEMD) was proposed by utilizing the frequency uniform distribution property of Gaussian white noise [14,15]. The EEMD algorithm is a noise assisted signal processing method; the essence of the algorithm is to perform multiple EMDs on the analyzed signal, superimposed with Gaussian white noise. Reconstruction errors can be reduced by increasing the ensemble size. By taking advantage of statistical characteristics of uniform distribution property of Gaussian white noise frequencies, the superimposed signal has continuity at different frequency scales. Thereby, EEMD can reduce the mode mixing problem of IMF components. An improved slope-based method was proposed to restrain the end effect, and such a method can decompose nonlinear time series into a set of IMFs efficiently and accurately [16]. Then, complementary ensemble empirical mode decomposition (CEEMD) was put forward to improve EEMD by adding positive and negative Gaussian white noise, and extracting the residue of added noise from the mixtures of signal and noise utilizing groups of complementary ensemble IMFs [17]. But for multivariate signals, EMD can only decompose each channel’s signal separately, resulting in different numbers and scales between multiple sets of IMFs. Such a phenomenon would be a disadvantage for subsequent synchronized correlation analysis, reducing fault diagnosis accuracy for multivariate signal [18]. During further research, bivariate empirical mode decomposition (BEMD) [19] was proposed to deal with bivariate signals. In the BEMD, projections of signals in a series of directions are employed to compute local means. It distributes direction vectors on a complex plane unevenly, and extreme values in the projection can be obtained by performing an interpolation using a complex spline function. Relevant researches about BEMD were conducted in fault diagnosis and condition monitoring [20]. Then trivariate empirical mode decomposed (TEMD) [21] were proposed, regarding trivariate signals as pure quaternions, and conduct signal projection in three-dimensional spaces and in a series of directions. Hence three-dimensional pure quaternion envelope curves can be obtained. After researches about BEMD and TEMD, aiming to analyze multivariate signal simultaneously, MEMD [22] was proposed as a significant approach in the field of signal processing. 

MEMD adopted real-valued projections on hyperspheres along multiple directions to compute the local means and envelopes. MEMD can decompose multivariate signal collected from different locations by multiple sensors into multiple sets of IMFs; IMF denotes same frequency locates in the same order, while overcoming the different numbers and scales between different sets of IMFs. This property is beneficial for simultaneous processing of multivariate signals in the field of fault diagnosis [23,24]. MEMD has also been successfully applied to many fields, such as biomedical signal processing [25], image processing [26], and fault diagnosis [27]. Due to the different locations of multiple sensors in rotating machinery, power imbalances usually exist among different channels of multi-sensor collected multivariate signals. The conventional projection method of multivariate signals having power imbalances between channels fails to obtain the desired effect. Thus adaptive-projection intrinsically transformed multivariate empirical mode decomposition (APIT-MEMD) [28] was proposed to deal with nonlinear and non-stationary signals, improving mode-mixing problems and obtaining fewer IMFs for multivariate signals. Most importantly, the method can accommodate power imbalance phenomena. Apart from these important benefits, the filter bank property of APIT-MEMD is also discussed in this paper, which is significant in multi-fault diagnosis. The filter bank properties of EMD [29], and MEMD [30], were studied in previous research, and having a dyadic filter bank property on per channel of multivariate signal when Gaussian white noise is present. The internal filter bank property of APIT-MEMD contributes to the separation of multi-faults of rolling bearings. The filter bank property of APIT-MEMD in the presence of Gaussian white noise is crucial for multivariate signal analysis. The extra noise channels can serve as references to enable more accurate and stable IMFs, which can also alleviate mode mixing problem.

Prior to finding fault frequencies in multivariate rolling bearing signals, fault numbers should be estimated. Singular value decomposition (SVD) [31] was the extension of spectral analysis on arbitrary matrix, and the obtained eigenvalues denotes the internal features of data. In the field of sources estimation in blind separation, SVD has been widely adopted to determine fault numbers, while eigenvalue decomposition and SVD with autocorrelation matrices was put forward to effectively reflect the internal features of data matrices [32,33,34,35]. Improved SVD was also proposed to estimate source numbers using dominant eigenvalues (DE) under low SNR conditions [36]. High order singular value decomposition (HOSVD) is a generalization of SVD, and internal property is a tensor decomposition. An analogy between multiple properties of the matrix was analyzed, and the relationship between third order tensor decomposition and matrix eigenvalue decomposition was discussed in previous research [37]. HOSVD can deal with third order tensors, constituted of matrices. It conducts Tucker decomposition towards constructed third order tensors, then finds its core tensor and conducts an SVD on all mode products [38]. The application of HOSVD appears widely in previous research. The third order tensor, namely high-dimensional data, can be constituted by IMFs of APIT-MEMD in this paper. Being an effective way to analyze high-dimensional data, tensors can preserve their intrinsic structure features [39]. The underlying feature of high dimensional data can be revealed by tensor decomposition in HOSVD, and reflected in eigenvalue values. It has been used in the field of blind source separation [40] and fault diagnosis [41]. 

This paper proposes a novel approach of multi-fault diagnoses of multivariate rolling bearing signal via APIT-MEMD and HOSVD. The approach utilizes APIT-MEMD to decompose multivariate signals into several IMF sets. By aligning IMF sets into a third order tensor, HOSVD is used to estimate the fault number with the assistance of a BIC [42,43]. Fault correlation factor (FCF) analysis [27] is used to determine the effective IMFs, and then to extract characteristic frequencies by spectrum analysis. The organization of this paper is as follows: Section 2 introduces the methodology of APIT-MEMD and HOSVD and illustrates the proposed approach; Section 3 presents the numerical simulations of APIT-MEMD and HOSVD, and comparisons between APIT-MEMD and MEMD, as well as HOSVD and SVD; Section 4 presents the application to practical multi-fault diagnoses of multivariate rolling bearing signals, in order to verify the effectiveness of the proposed approach in this paper. The conclusions of the research are given in Section 5.

## 2. Methodology

### 2.1. Adaptive-Projection Intrinsically Transformed Multivariate Empirical Mode Decomposition

In the field of signal processing, the MEMD is the extension of EMD and can decompose multivariate signal into a series of IMF sets. Each IMF set has the same length, and components containing the same frequencies are organised in the same order. The detailed algorithm of MEMD has been elaborated in the previous work of authors [27]. To elaborate the principle of APIT-MEMD, non-uniformly sampled trivariate empirical mode decomposition (NS-TEMD) is described here as an illustration [44]. The NS-TEMD algorithm is basically about improving the standard TEMD [21] by relocating the direction vectors adopting conventional uniform sampling method to new locations on an ellipsoid. The eigenvectors and eigenvalues of the covariance matrix are used to determine the direction and relative powers. As for multivariate signals, APIT-MEMD adopts similar strategy by relocating *n*-dimensional uniform vectors on *n*-dimensional ellipsoids [28]. The scheme of the proposed method is complicated, owing to the reason that global highest curvature does not always correspond to the local principle component direction. Such disagreement would cause suboptimal local mean estimation, which is the crucial part in MEMD.

The APIT-MEMD adopts the following projection strategy. The first principle component direction represents the largest power imbalance and determines the correlation in different channels of original multivariate signals adaptively. For instance, assuming there is a multivariate signal ***s***(*t*), and its covariance matrix is **C** = *E*{***s***(*t*)***s***(*t*)*^T^*} (*E* is statistical expectation operator). The first principle component direction can be determined using the covariance matrix eigendecomposition as follows.

(1) $$C=\Sigma \Lambda \Sigma^{T}$$

where the **Σ** = [**Σ**_1_, **Σ**_2_, …, **Σ***_n_*] denotes eigenvectors matrix, and all values on diagonal matrix **Λ** = diag{*λ*_1_, *λ*_2_, …, *λ_n_*} are eigenvalues of **C**. The largest eigenvalue corresponds to eigenvector **Σ**_1_, which is the first principal component direction, namely points in the largest power imbalance direction. **Σ**_1_ can be used to construct another vector **Σ***_o_*_1_ along its opposite direction diametrically, namely **Σ***_o_*_1_ = −**Σ**_1_. Subsequently **Σ**_1_ and **Σ***_o_*_1_ are employed to relocate all direction vectors generated by uniform projection schemes previously. During the sifting process, multivariate sifting inputs are projected along these adaptive direction vectors, and local means are estimated on basis of conventional MEMD [22]. The proposed APIT-MEMD can produce fewer IMFs, while exhibiting better performance than MEMD, because of the large number of adaptive projection vectors that are previously obtained [28]. The procedures of proposed APIT-MEMD are given in Table 1.

**Figure 1:**
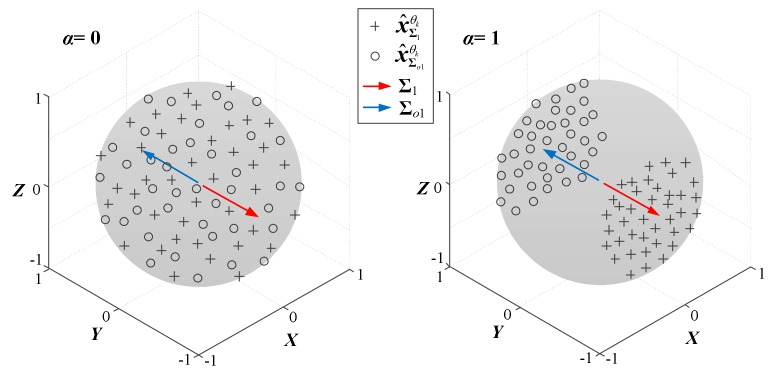
Adaptive projection vectors by adopting the proposed strategy (*α* = 0, 1).

The *α* values are determined by the degree of power imbalances between different channels of a multivariate signal. In the 5th step of detailed procedures of APIT-MEMD, *α* = 0 denotes that there are no accountable power imbalances; conversely, *α* = 1 denotes high power imbalances between various channels. The illustration of *α* is shown in Figure 1. For the situation with power imbalances between channels in multivariate signal processing in practical situations, APIT-MEMD is very suitable for dealing with the sampling of multivariate signal. The adaptive projection strategy would generate much more adaptive projection vectors, thus exhibiting better performance.

As for multi-fault diagnoses of multivariate rolling bearing signals, the filter bank property of APIT-MEMD in the presence of Gaussian white noise is discussed hereafter in this paper. In previous research, MEMD has been verified as having the properties of a dyadic filter bank on per channel of multivariate signals, and can arrange corresponding IMFs from various channels to the same frequency ranges. The obtained filtered IMFs represent the same frequencies, present in the same orders. The filter bank property of APIT-MEMD, in the presence of Gaussian white noise, is crucial for multivariate signal analysis. The extra noise channels can serve as references to enable more accurate and stable IMFs, which can also alleviate mode mixing problem. A graphical illustration of employing the filter bank property will be given in 2.3, along with the graphical illustration of the proposed multi-fault diagnosis scheme, in order to better explain the entire approach. The properties of APIT-MEMD will be discussed and verified by numerical simulations, including comparison with MEMD in Section 3. The procedures of proposed APIT-MEMD are given in Table 2.

Based on the above discussion, it can be concluded that APIT-MEMD has three significant properties, which can play important roles in multi-fault diagnoses of rotating machinery. The three properties are summarized as follows:

- APIT-MEMD can align IMFs from multiple channels to the same frequency ranges, just like MEMD, and the same characteristic frequencies locate in the same orders respectively.
- APIT-MEMD can generate large numbers of adaptive projection vectors to alleviate the adverse effect of power imbalances between channels, among multi-sensor signal acquisition systems.
- APIT-MEMD has a filter bank property in the presence of Gaussian white noise; the extra noise channels can serve as references to enable more accurate and stable IMFs, in order to alleviate mode mixing problem.

### 2.2. High Order Singular Value Decomposition

The application of HOSVD in third order tensors has been examined in previous research [38]. Here HOSVD is adopted to compute the optimal eigenvalues of third order tensors constructed by IMFs matrices. The methodology of HOSVD is present in this section. The SVD of a matrix is depicted here briefly to explain the principle of HOSVD hereafter. Assuming **X** is a matrix of *m* × *n*, and it can be decomposed by SVD into three matrices as follows:

(2) $$X=U\Lambda V^{T}$$

where **U** and **V*^T^*** are unitary matrices of *m* × *m* and *n* × *n* respectively, and span the row and column space of **X** respectively. **Λ** is a diagonal matrix of *m* × *n*, and all values on diagonal are eigenvalues of **X**. All eigenvectors of **U** are orthonormal to each other; the same may be said of **V**, so SVD can present an orthonormal coordinate system for **X** by spanning the spaces [37]. **X** can also be presented regard to *k*-mode products as follows.

(3) $$X=(\Lambda )\times_{1}(U)\times_{2}(V)$$

where ×*_k_* means *k*-mode product, and *k* = 1, 2. 

**Figure 2:**
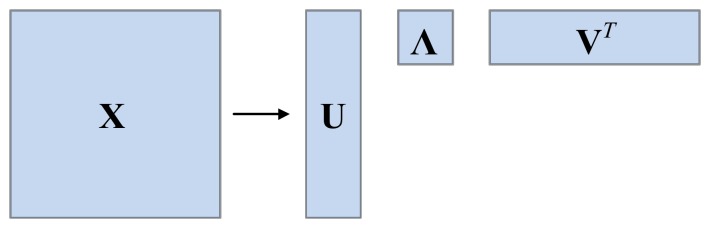
Illustration of SVD approximation.

**Figure 3:**
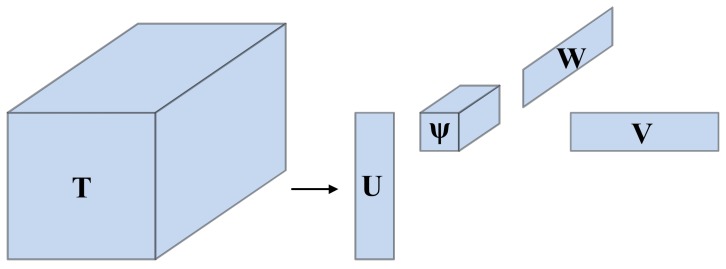
Illustration of HOSVD approximation.

The HOSVD is an extension of SVD. HOSVD conducts Tucker decomposition towards constructed third order tensors, then finds its core tensor and conducts an SVD on all mode products separately [38]. *N* dimensional data can construct *N*th order tensors that have *N* indices. A third tensor constructed by IMFs matrices is analyzed in this paper as an example. A third order tensor can be decomposed by Tucker decomposition as follows:

(4) $$T=(\psi )\times_{1}(U)\times_{2}(V)\times_{3}(W)$$

where **ψ** represents core tensor, while **U**, **V**, **W**, are orthogonal matrices respectively. Likewise, ×*_k_* indicates *k*-mode product. The diagram illustration of SVD and HOSVD are shown in Figure 2 and Figure 3 respectively. 

Matricizing of a tensor can be understood as rearranging the elements of the tensor. The *N*-mode matricizing of a tensor **T** means converting the tensor into matrices based on its dimension *N*, signified as **T**_(*N*)_, and *N* = 1, 2, 3. As for the third order tensors in this paper, the HOSVD of **T** can be conducted through obtaining the orthogonal matrices **U**, **V**, **W** respectively. These three matrices are the left singular matrices of **T**_(*N*)_ (*N* = 1, 2, 3); they can be obtained by computing SVDs three times on all mode matricizing of **T** respectively.

(5) $$\begin{array}{l} T_{\left(1\right)}=U\Lambda_{1}{\left(R_{1}\right)}^{T}\\ T_{\left(2\right)}=U\Lambda_{2}{\left(R_{2}\right)}^{T}\\ T_{\left(3\right)}=U\Lambda_{3}{\left(R_{3}\right)}^{T} \end{array}$$

where **R**_(*N*)_ are right singular matrices of **T**_(*N*)_, *N* = 1, 2, 3. Then the core tensor **ψ** can be computed as follows.

(6) $$\psi =(T)\times_{1}(U^{T})\times_{2}(V^{T})\times_{3}(W^{T})$$

In the end, the basis matrices **X***_i_* of tensor **T** can be represented as:

(7) $$X_{i}=[\psi (:,:,i)]\times_{1}(U)\times_{2}(V)$$

where *i* = 1, 2, …, *M*, and *M* is the number of IMFs obtained by APIT-MEMD. Owing to the orthogonality of tensor core **ψ**, **X***_i_* (*i* = 1, 2, …, *M*) are orthogonal to each other, and also meet the flowing situation.

(8) $$\langle X_{i},X_{j}\rangle =trace[{\left(X_{i}\right)}^{T}X_{j}]=0$$

where *i* ≠ *j*, and *i*, *j* = 1, 2, …, *M*. Regard to these basis matrices, all singular values are non-negative here, and can be obtained as follows.

(9) $$\sigma_{i}={\left\|\psi (:,:,i)\right\|}_{F}$$

where *i* = 1, 2, …, *M*, and $\sigma_{1}\geq \sigma_{2}\geq \cdots \geq \sigma_{N}\geq 0$. Thus far, the singular values of third order tensor constructed by IMFs matrices are computed, which are obtained by APIT-MEMD of multivariate rolling bearing signals. 

### 2.3. The Proposed Novel Multi-Fault Diagnosis Approach via APIT-MEMD and HOSVD

**Figure 4:**
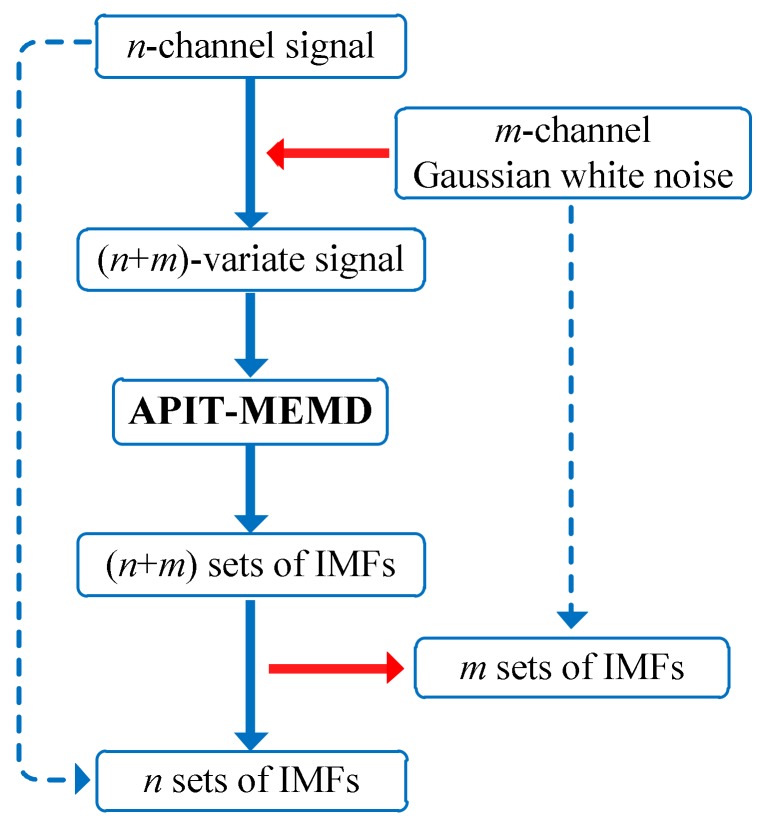
The schematic diagram of employing filter bank property.

**Figure 5:**
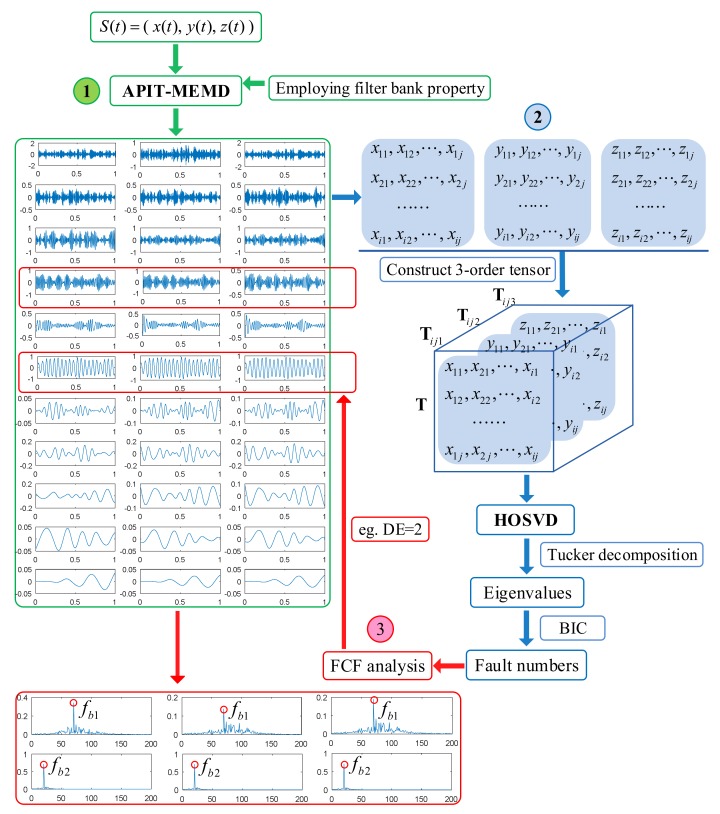
The proposed multi-fault diagnosis scheme of multivariate rolling bearing signal.

Using multiple sensors to conduct signal acquisition can yield more fault characteristic information. Then the multivariate signal can be used for fault diagnosis; the multi-fault situation is discussed in this paper. The number of faults is required to be estimated before conducting fault diagnosis. Here APIT-MEMD is used to decompose multivariate signal into multiple IMF groups. Then, by aligning all IMF groups to the third order tensor, HOSVD can be conducted. The obtained optimal singular values are determined by BIC, and can be used to estimate fault numbers. Hence the number of DE can be determined. Afterwards, FCF analysis [27] is conducted to determine the orders of effective IMFs, and then to achieve multi-fault diagnoses of multivariate rolling bearing signals. The schematic diagram of employing filter bank properties is illustrated in Figure 4. The scheme of the proposed method in this paper is illustrated in Figure 5.

## 3. Numerical Simulations

### 3.1. Numerical Simulation of EEMD

**Figure 6:**
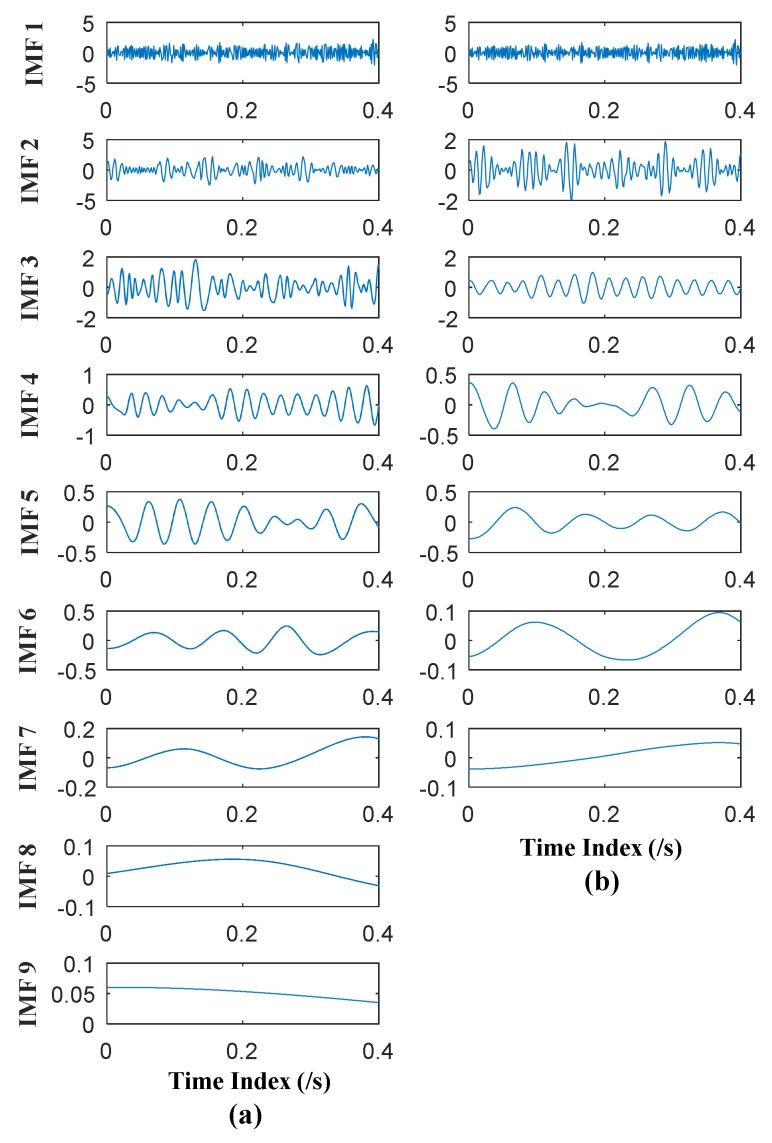
(**a**) Time domain plots of IMFs of EMD; (**b**) Time domain plots of IMFs of EEMD.

As for a single sensor system, the filter bank properties of EEMD in the presence of Gaussian white noise are illustrated here, due to this is the essence of the filter bank properties of APIT-MEMD in the presence of Gaussian white noise. The filter bank property of EEMD and APIT-MEMD, by utilizing frequency uniformly distribution property of Gaussian white noise, is of great importance in alleviating or solving mode mixing problem. The collected signal of faulty bearings usually has additive noise, and Gaussian white noise is used to simulate a practical situation [45]. When outer ring, inner ring, or rolling element faults appear in rolling bearing, the characteristic frequencies can be found in its frequency domain plot. The vibration signal of faulty rolling bearings can be simulated by a simplified signal model, [46] as follows:

(10) $$x(t)=\alpha \sin(2\pi f_{b}t)[1+\beta \cos(2\pi f_{r}t)]$$

where *f_b_* denotes the fault characteristic frequency of the rolling bearing, and *f_r_* denotes the rotational frequency. *α* and *β* denote the power size. The sampling point is 1024, and the sampling frequency is 1024 Hz. *s* denotes the Gaussian white noise with variance of 1 and a mean of 0. The simulated faulty rolling bearing signal is as follows.

(11) $$x_{1}(t)=\sin(2\pi f_{1}t)+\cos(2\pi f_{2}t)[1+\sin(2\pi f_{3}t)]+s$$

where *f*_1_ = 40 Hz, *f*_2_ = 90 Hz, *f*_3_ = 15 Hz. EMD and EEMD are then conducted respectively on the above signal. In EEMD, Gaussian white noise of the same length is added to the signal; the ensemble size was set at 20. The time domain plots of obtained IMFs of EMD and EEMD are shown in Figure 6.

**Figure 7:**
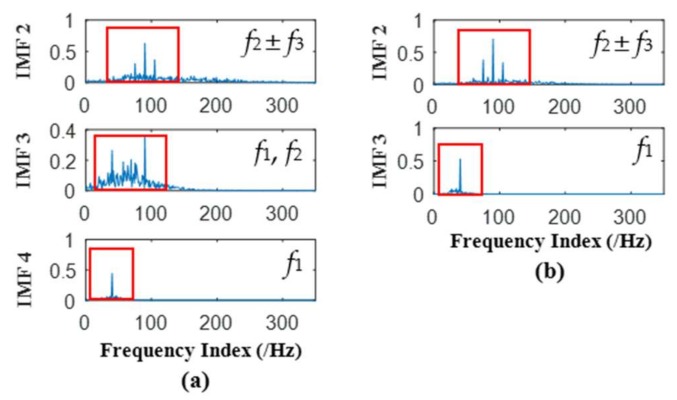
(**a**) Frequency domain plots of IMFs of EMD; (**b**) Frequency domain plots of IMFs of EEMD.

It can be seen from Figure 6 that 9 orders of IMFs are obtained by EMD, while 7 orders of IMFs are obtained by EEMD. Obviously EEMD can generate fewer orders of IMFs than EMD. Further, to measure the effectiveness of EEMD in alleviating mode mixing problem, during extraction of accurate characteristic frequencies of the simulated signal, correlation analysis is employed. The correlation analysis can be used to find effective IMFs; a similar method was elaborated in author’s previous research [27]. During correlation analyses, the 2nd, 3rd and 4th IMFs of EMD are effective, while 2nd and 3rd IMFs of EEMD are effective. The frequency domain plots of effective IMFs of EMD and EEMD are shown in Figure 7.

It can be seen from Figure 7a that characteristic frequencies *f*_2_ ± *f*_3_ appear in 2nd IMF of EMD, *f*_1_ appears in 4th IMF of EMD, but *f*_1_ and *f*_2_ both appear in 3rd IMF of EMD, which is due to the phenomenon of mode mixing, along with many additive noise frequencies. While it can be seen from Figure 7b that characteristic frequencies *f*_2_ ± *f*_3_ appear in 2nd IMF of EEMD, *f*_1_ appears in the 4th IMF of EEMD, and both characteristic frequencies are very clear. There is no mode mixing problem in the obtained IMFs of EEMD. So as for one channel simulated rolling bearing signal, it can be seen EEMD is superior to EMD in enabling more accurate and clear IMFs, and in alleviating mode mixing problem. The filter bank property of APIT-MEMD in the presence of Gaussian white noise is derived from the essence of EEMD; the following Section 3.2 can verify this property of APIT-MEMD, along with the other two properties of APIT-MEMD. 

### 3.2. Numerical Simulation of APIT-MEMD

As for a multiple sensor system, original components are collected by multiple sensors simultaneously, and each collected signal is composed of all original components. Here a three-channel signal with additive Gaussian white noise is adopted for numerical simulation, and power imbalances exist between channels. The sampling point is 1024, and sampling frequency is 1024 Hz. *s* denotes Gaussian white noise with variance of 1 and mean of 0. The simulated faulty rolling bearing signals are composed of original components, and the components are as follows.

(12) $$\begin{array}{l} x_{1}(t)=\sin(2\pi f_{1}t)[1+\cos(2\pi f_{2}t)]\\ x_{2}(t)=\sin(2\pi f_{3}t)\\ x_{3}(t)=\cos(2\pi f_{4}t)[1+\sin((2\pi f_{5}t))] \end{array}$$

where *f*_1_ = 30 Hz, *f*_2_ = 10 Hz, *f*_3_ = 70 Hz, *f*_4_ = 120 Hz, *f*_5_ = 15 Hz. To simulate the power imbalances between different channels, different amplitudes are set to simulated signals, owing to the fact that the amplitudes of signals from different channels can depict different magnitudes of power. For the 1st channel, the amplitude is set as 1, similarly, 3 for 2nd channel, and 6 for 3rd channel. The resulting trivariate signal *S*(*t*) is as follows.

(13) $$\begin{array}{l} S_{1}(t)=x_{1}(t)+x_{2}(t)+x_{3}(t)+s\\ S_{2}(t)=3x_{1}(t)+3x_{2}(t)+3x_{3}(t)+3s\\ S_{3}(t)=6x_{1}(t)+6x_{2}(t)+6x_{3}(t)+6s \end{array}$$

**Figure 8:**
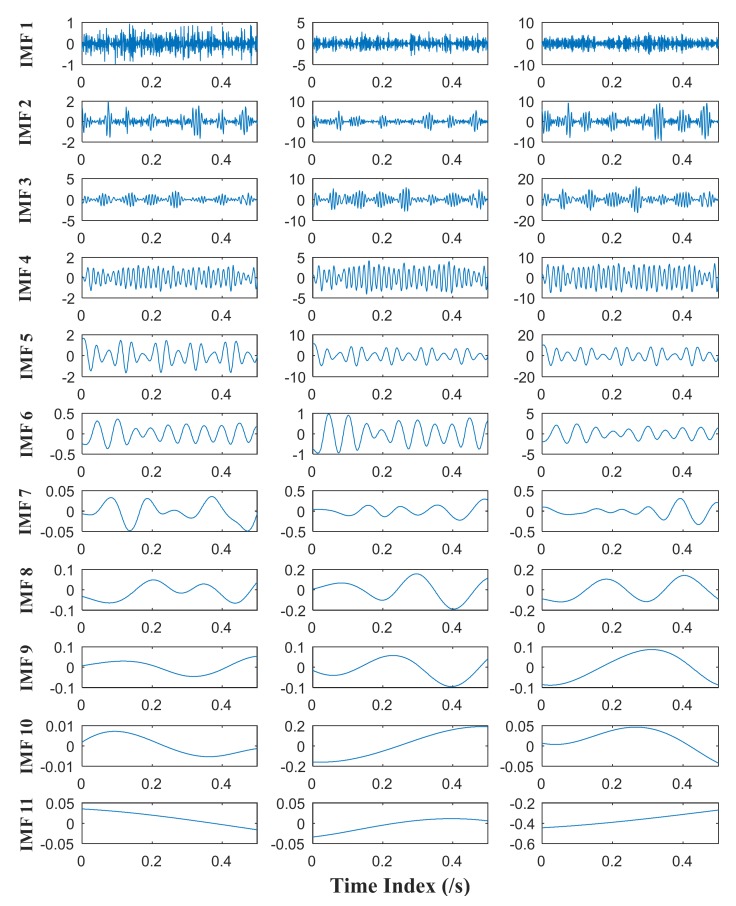
Time domain plots of IMFs of MEMD.

**Figure 9:**
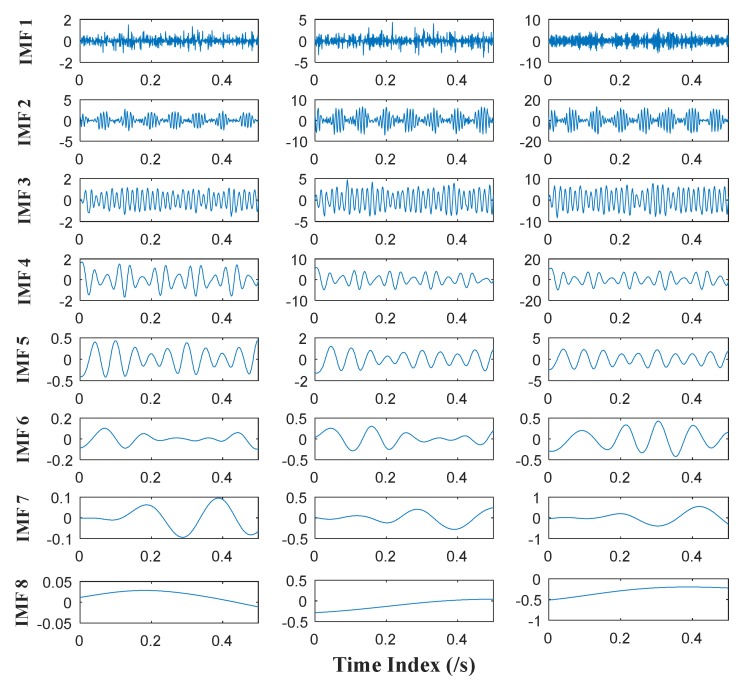
Time domain plots of IMFs of APIT-MEMD.

MEMD and APIT-MEMD respectively are then conducted on the above trivariate signal. In APIT-MEMD, *α* is chosen as 1, and two-channel Gaussian white noise of the same length is added to the trivariate signal; thus five sets of IMFs are obtained at the end of the numerical simulation. In this way, the filter bank property of APIT-MEMD can be utilized. Then two sets of IMFs corresponding to two-channel Gaussian white noise are discarded from the whole set. The time domain plots of obtained IMFs of MEMD and APIT-MEMD are shown in Figure 8 and Figure 9 respectively. 

**Figure 10:**
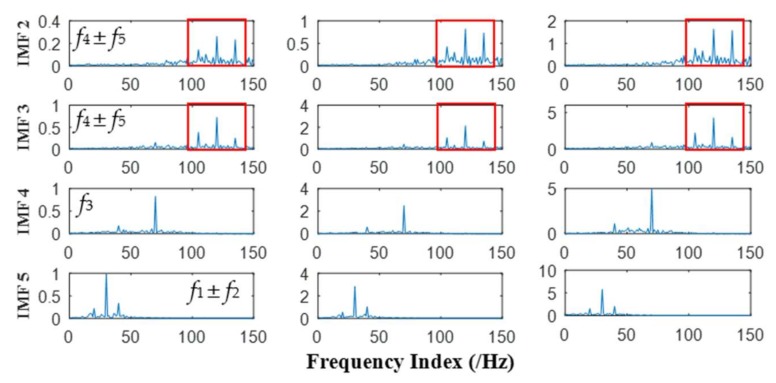
Frequency domain plots of effective IMFs of MEMD.

**Figure 11:**
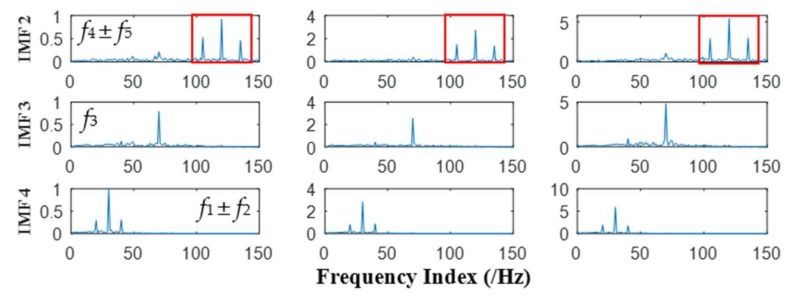
Frequency domain plots of effective IMFs of APIT-MEMD.

**Figure 12:**
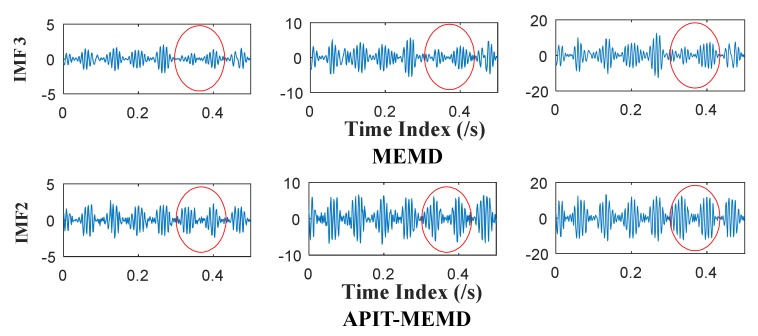
Selected time domain plots of IMFs of MEMD and APIT-MEMD.

It can be seen from Figure 8 and Figure 9 that 11 orders of IMFs are obtained by MEMD, while 8 orders of IMFs are obtained by APIT-MEMD. Obviously APIT-MEMD can generate fewer orders of IMFs than MEMD. Further, to measure the effectiveness of the proposed method in extracting accurate characteristic frequencies of the simulated collected trivariate signal, FCF analysis is employed to find the effective IMFs; such a method was proposed and elaborated in author’s previous research [27]. During the FCF analysis, 2nd, 3rd, 4th, and 5th IMFs of MEMD are effective, and frequency domain plots of them are shown in Figure 10. 2nd, 3rd, and 4th IMFs of APIT-MEMD are effective, and frequency domain plots of them are shown in Figure 11. The time domain plots of 3rd IMFs of MEMD and 2nd IMFs of APIT-MEMD are shown in Figure 12.

It can be seen from Figure 10 that the characteristic frequencies of *x*_3_(*t*) appear both in the 2nd and 3rd IMFs of MEMD, along with additive noise frequencies. While it can be seen from Figure 11 that 2nd IMFs of APIT-MEMD denote clear characteristic frequencies of *x*_3_(*t*), and have less noise and greater amplitudes. As for IMFs denoting the characteristic frequencies of *x*_1_(*t*), 4th IMFs of APIT-MEMD are clearer than 5th IMFs of MEMD in terms of side frequencies. It can also be seen from the time domain plots shown in Figure 12 that the amplitudes of IMFs of APIT-MEMD are greater than those of the MEMD, and that waveforms are clearer too. It should be noted that *x*_3_(*t*) and *x*_1_(*t*) are derived from rolling bearing models. So, as for simulated rolling bearing signals, it can be seen that APIT-MEMD is superior to MEMD in enabling more accurate and clear IMFs, and alleviating mode mixing problem.

Due to the numerical simulation of APIT-MEMD of multivariate signal and the comparison to MEMD, the properties of APIT-MEMD discussed in Section 2.1 are verified here. It can be concluded that the APIT-MEMD can align IMFs from multiple channels to the same frequency ranges as those of the MEMD. APIT-MEMD has better performance than MEMD by generating adaptive projection vectors, and employing the filter bank property in the presence of Gaussian white noise. 

### 3.3. Numerical Simulation of HOSVD 

**Figure 13:**
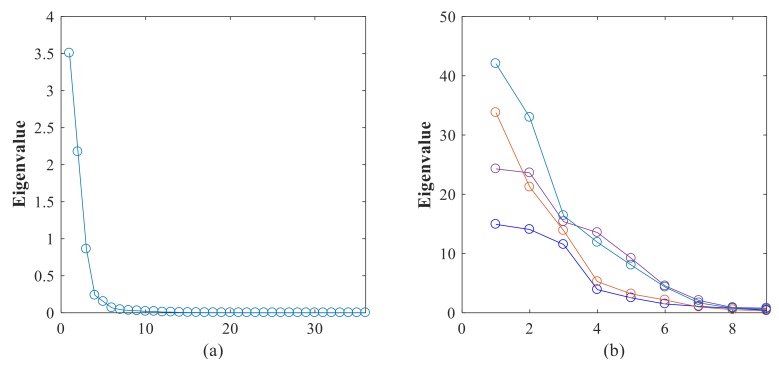
(**a**) Eigenvalues obtained by HOSVD; (**b**) Four sets of eigenvalues obtained by SVD.

Among multi-sensor signal acquisition systems, each channel signal is the combination of all components with different degrees of energy. SVD is usually adopted to compute eigenvalues of a single channel signal to estimate the fault number in a blind separation. The estimation is a challenge because of the limited information available for a single channel signal. The proposed method in this paper uses multiple sensors to collect multivariate signals, so instead of conducting SVD on each channel signal, it is is proposed that HOSVD be conducted on the multivariate signal. By this means, one may estimate the fault number among multi-sensor signal acquisition system. Theoretically, HOSVD can better explore underlying information by making use of all channel signals. To simulate real situations, here multivariate signal *S*(*t*), composed of three simulated fault signals, is used to verify the proposed method. Gaussian white noises are also added into each channel to monitor background noise in rotating machinery. *x*_1_(*t*), *x*_2_(*t*), *x*_3_(*t*) are simulated faulty rolling bearing signals, and *S*(*t*) is four-channel signal composed of *x*_1_(*t*), *x*_2_(*t*), *x*_3_(*t*), random matrix **A** is adopted to simulate multi-sensor situation; *s* denotes the Gaussian white noise with variance of 1, and mean of 0.

(14) $$\begin{array}{l} x_{1}(t)=\sin(2\pi f_{b1}t)[1+\cos(2\pi f_{r1}t)]\\ x_{2}(t)=2\cos(2\pi f_{b2}t)[1+0.5\sin(2\pi f_{r2}t)]\\ x_{3}(t)=\sin(2\pi f_{b3}t)[1+\cos(2\pi f_{r3}t)] \end{array}$$

(15) $$\left(\begin{array}{l} S_{1}(t)\\ S_{2}(t)\\ S_{3}(t)\\ S_{4}(t) \end{array}\right)=A\left(\begin{array}{l} x_{1}(t)\\ x_{2}(t)\\ x_{3}(t) \end{array}\right)+\left(\begin{array}{l} s\\ s\\ s\\ s \end{array}\right),\;\mathrm{and}\;A=\mathrm{random}(4,3)$$

where *f_b_*_1_ = 20 Hz, *f_b_*_2_ = 70 Hz, *f_b_*_3_ = 150 Hz, *f_r_*_1_ = 10 Hz, *f_r_*_2_ = 8 Hz, *f_r_*_3_ = 10 Hz. Then after conducting APIT-MEMD, four IMF groups can be obtained. Then, all IMF groups are aligned according to the scheme in Figure 5 in Section 2.3, in order to construct third order tensor **T**. Here a comparison of SVD and HOSVD is given to elaborate the effectiveness of the proposed method. Based on traditional methods, SVD is conducted on the covariance matrix of transposed matrix composed of each IMF group. HOSVD is then conducted on the third order tensor **T**. The eigenvalues obtained from HOSVD and SVD are shown in Figure 13. 36 eigenvalues are obtained by HOSVD, and four sets of eigenvalues are obtained by SVD; each set has 9 eigenvalues.

From the eigenvalues obtained above, it can be seen from the results of HOSVD of the constructed tensor that the first 3 eigenvalues are dominated, while from the results of SVD of each channel, dominated eigenvalues cannot be found. BIC is then used to estimate the dimension of a noisy signal subspace, which is the fault number of original signal here. Research of BIC in blind source separation has been conducted in previous work by the authors [42]. Here the BIC values of eigenvalues obtained by HOSVD reach a maximum when *k* is 3, namely the fault number is estimated at 3. As the BIC values of eigenvalues obtained by SVD of each channel are disordered, no clear maximum value can be found. Several numerical simulations were conducted, and led to similar results with the proposed method. It can be concluded that HOSVD is superior than SVD when dealing with multivariate signal containing multi-fault resources. 

## 4. Application Researches

**Figure 14:**
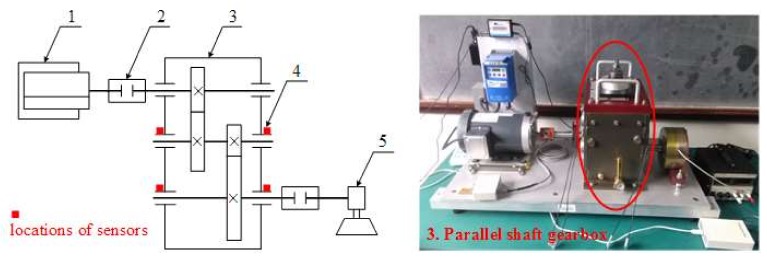
The schematic diagram and the picture of the experimental apparatus. 1-Variable speed drive, 2-Torque transducer and encoder, 3-Parallel shaft gearbox, 4-Test points, 5-Programmable magnetic brake.

**Figure 15:**
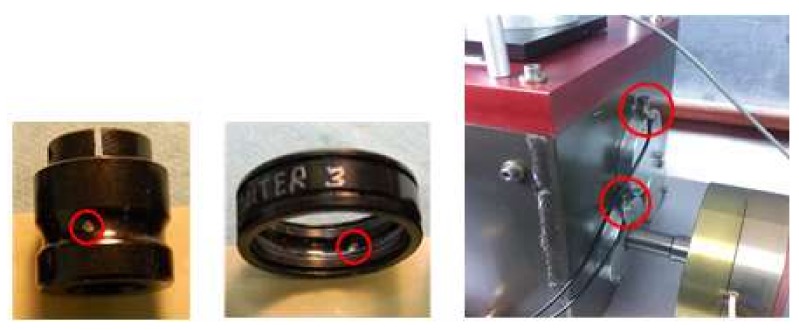
The IRF and ORF rolling bearings, and two sensors of one side.

To further validate the effectiveness of the proposed method in the application of multi-fault diagnosis, the experiment was conducted on Drivetrain Diagnostics Simulator. The experimental apparatus was produced by SQI Company, United States. The experimental apparatus consisted of a variable speed drive, a torque transducer and encoder, a parallel shaft gearbox, and a programmable magnetic brake. The experiment was conducted to collect multivariate rolling bearing signals; among the four rolling bearings in the parallel shaft gearbox, one has an inner ring defect and another has an outer ring defect. The sensors are placed on four rolling bearing end plates to collect acceleration signals. The schematic diagram of the apparatus is shown in Figure 14, along with its photo. The faulty inner and outer rings and two sensors of one side are shown in Figure 15.

The experiment was conducted to collect the vibration signal of weak fault in the rolling bearing. During the experiment, the sampling frequency was 8192 Hz, the sampling point was 8192, and the rotational frequency *f_r_* was set at 15 Hz. The fault characteristic frequencies of experimental FAFNIR deep groove rolling bearing were computed and are shown in Table 3. 

**Figure 16:**
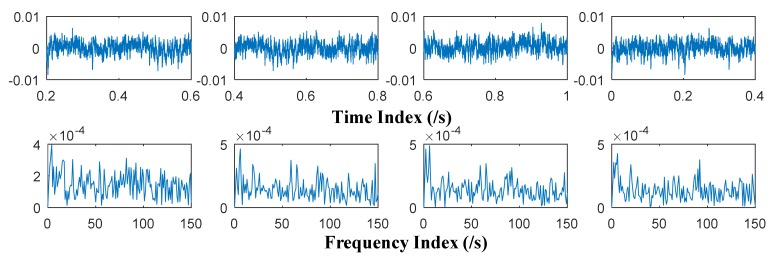
Time and frequency domain plots of collected multivariate signal.

The time and frequency domain plots of the collected multi-fault multivariate rolling bearing signal are shown in Figure 16.

**Figure 17:**
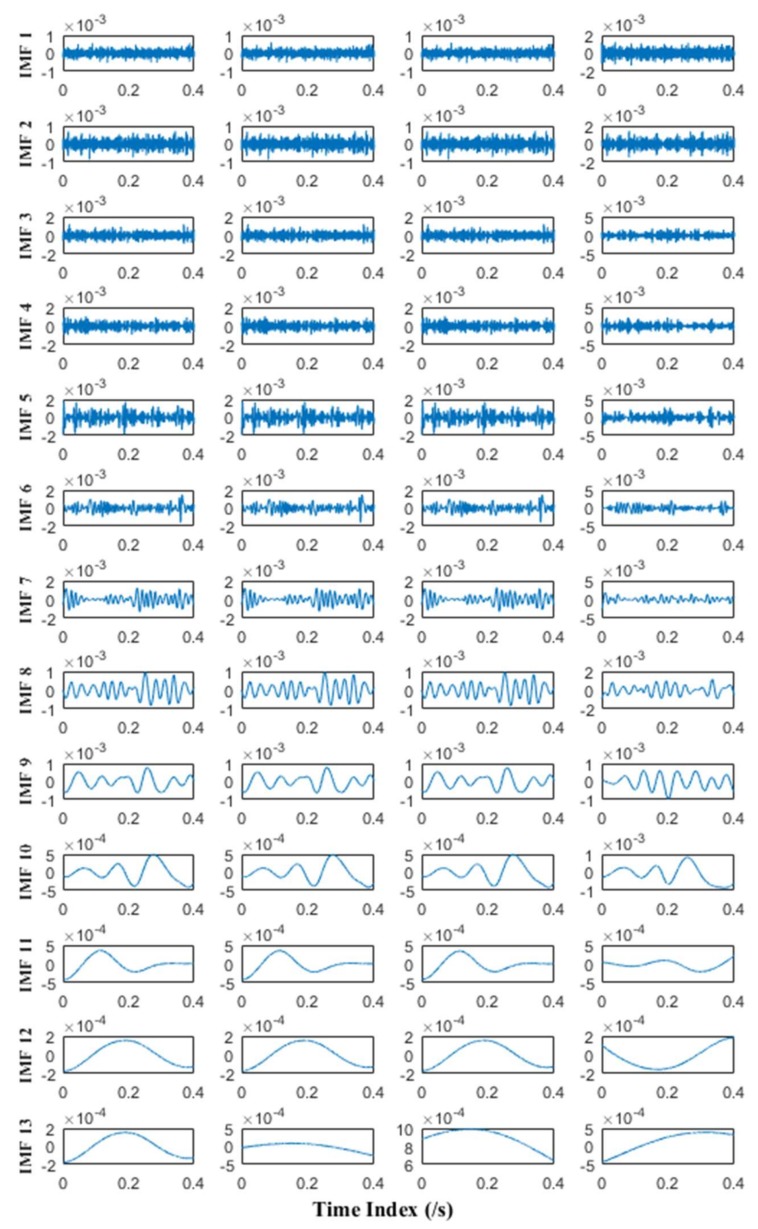
Time domain plots of IMFs of MEMD.

**Figure 18:**
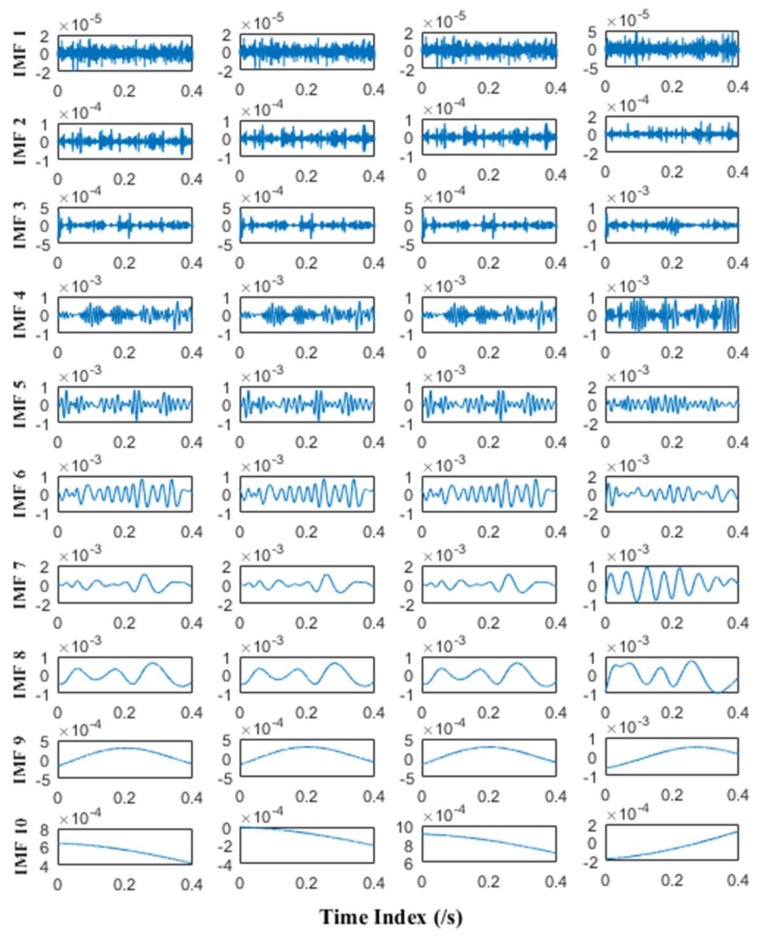
Time domain plots of IMFs of APIT-MEMD.

Prior to conducting MEMD and APIT-MEMD, the NLM denoising method is used to denoise the multivariate signal for preprocessing. The NLM method has been studied in previous research by the authors [47]. MEMD and APIT-MEMD were then conducted respectively on the above multivariate signal. In APIT-MEMD, *α* was chosen as 0.5, and two-channel Gaussian white noise with same length were added to the multivariate signal to employ the filter bank property of APIT-MEMD. Afterwards, two sets of IMFs corresponding to two-channel Gaussian white noise were discarded from the whole sets. The time domain plots of obtained IMFs of MEMD and APIT-MEMD are shown in Figure 17 and Figure 18 respectively. 

**Figure 19:**
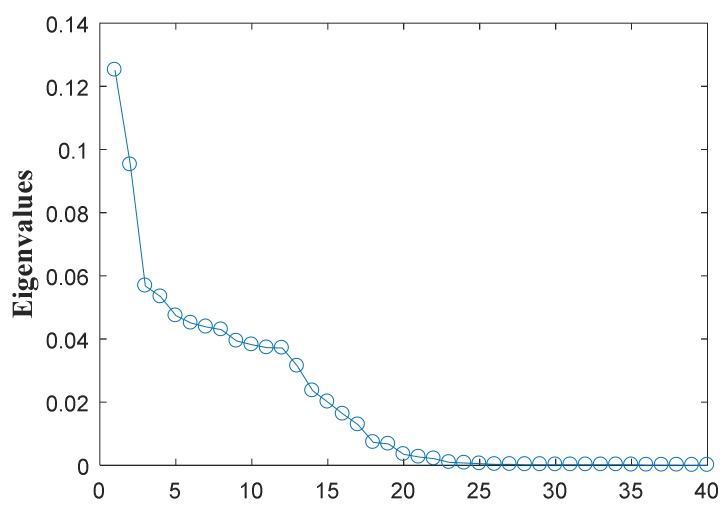
Eigenvalues obtained by HOSVD.

It can be seen APIT-MEMD generates 10 IMF sets, while MEMD generates 13 IMF sets. Namely APIT-MEMD can generate fewer orders of IMF sets, owing to the fact that APIT-MEMD can alleviate power imbalance between four channels and the filter bank property, to ease mode mixing problem. All IMF groups were aligned according to the scheme in Figure 5 in Section 2.3, to construct third order tensor **T**. The eigenvalues obtained from HOSVD are shown in Figure 19.

From the eigenvalues obtained above, it can be seen from the results of the HOSVD of the constructed tensor that the first 2 eigenvalues are dominated. BIC [42] was then used to estimate the dimension of noisy signal subspace, which is the fault number of original signal here. Here the BIC values of eigenvalues obtained by HOSVD reached a maximum when the *k* was 2, namely, the fault number is estimated at 2. Afterwards, FCF analysis [27] was conducted to obtain the effective IMFs of APIT-MEMD. Two order IMFs needed to be selected for multi-fault diagnosis of multivariate rolling bearing signal. The FCF of each order of IMF sets were computed and are shown in Table 4.

**Figure 20:**
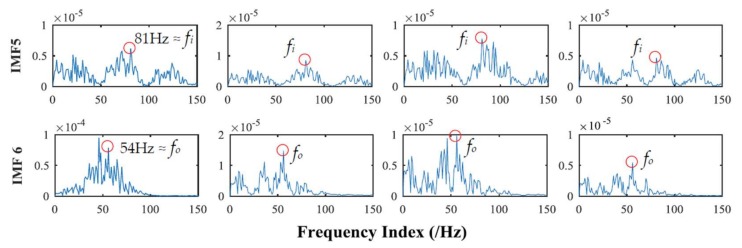
Frequency domain plots of effective IMFs of MEMD.

**Figure 21:**
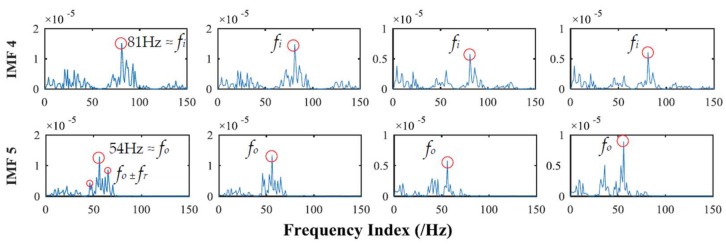
Frequency domain plots of effective IMFs of APIT-MEMD.

The correlation between the specific order of IMF sets and the collected multivariate rolling bearing signal are shown in Table 4. The larger FCF values mean that the greater the correlation, the maximum 2 values locate in 4th and 5th order IMF. Similarly, 5th and 6th order of IMF sets obtained by MEMD are selected to be compared with the results of APIT-MEMD. The frequency domain plots of effective IMFs of MEMD and APIT-MEMD are shown in Figure 20 and Figure 21 respectively.

In the field of multi-fault diagnosis of rolling bearings, fault feature extraction is more complicated than single fault situations. Hence, mode mixing problems are more serious than single fault diagnoses. In this paper, the multivariate signal was collected (rather than that of a single channel), so power imbalance between channels was inevitable. So theoretically, APIT-MEMD would extract more accurate characteristic frequencies than MEMD. In the process of obtaining frequency domain plots, the frequency resolution equals the result of sampling frequency divided by sampling point, thus the frequency resolution in this paper is 1 Hz. Faulty frequencies of rolling bearings are obtained by empirical computing equations [27] for bearing fault frequencies, so the extracted frequencies cannot be precisely 81.45 Hz and 53.58 Hz. Therefore, the extracted 81 Hz and 54 Hz can respectively represent the inner ring fault and outer ring fault characteristic frequencies *f_i_* and *f_o_* in this paper. It can be seen from Figure 20 that the fault frequencies of both the inner and outer race faults cannot be extracted accurately by MEMD, as *f_i_* and *f_o_* tend to be overwhelmed by other noise frequencies. While in Figure 21, the fault frequencies of both inner and outer race faults were extracted clearly. In the 5th order of effective IMFs of APIT-MEMD, side frequencies *f_o_* ± *f_r_* can also be identified. Therefore, the effectiveness of the proposed multi-fault diagnoses of multivariate rolling bearing signal via APIT-MEMD and HOSVD has been verified by practical application. 

## 5. Conclusions

In this paper, the research work elaborated the effectiveness of the proposed multi-fault diagnosis of multivariate rolling bearing signal via APIT-MEMD and HOSVD. Theoretical derivation demonstrates the significance of the proposed approach, and its effectiveness is verified by numerical simulations and practical application. The research work in this paper clearly indicates that APIT-MEMD can not only align IMFs from multiple channels to the same frequency ranges same as those of the MEMD, but also can alleviate the adverse effect of power imbalances between channels, and ease mode mixing problem by employing the filter bank property. In the processing of multivariate rolling bearing signal containing multi-fault, the inner and outer race faulty characteristic frequencies can be extracted correctly and clearly by APIT-MEMD, while MEMD cannot achieve such a result. By comparison with MEMD, the APIT-MEMD can be verified as being superior to MEMD. By constructing the IMF sets to a tensor, HOSVD can deal with multivariate signals, and estimate fault numbers by analyzing the obtained eigenvalues. The achieved result is better than SVD by processing each channel signal. The numerical simulation also demonstrates the effectiveness of HOSVD. Hence it can be concluded that the proposed approach is promising in the field of multi-fault diagnosis of multivariate rolling bearing signal, and has real significance in structural health monitoring. 

## Figures and Tables

**Table 1:** The detailed procedures of APIT-MEMD.

(1) Given an *n*-variate signal *s*(*t*), and its covariance matrix C is performed eigendecomposition, C = ΣΛΣ*^T^*, Σ denotes the eigenvector matrix, and Λ denotes the eigenvalues matrix. The largest eigenvalue *λ*_1_ corresponds to eigenvector Σ_1_, which is the first principal component. (2) Construct another vector Σ*_o_*_1_ along the opposite direction of Σ_1_ diametrically. (3) Using Hammerseley sequence, uniformly sample an (*n*−1) sphere to obtain *K* direction uniform projection vectors ${\left\{x^{\theta_{k}}\right\}}_{k=1}^{K}$. Then compute Euclidean distances of each direction vector to Σ_1_. (4) Relocate half of the uniform projection vectors $x_{\Sigma_{1}}^{\theta_{k}}$, which are near to Σ_1_, using ${\hat{x}}_{\Sigma_{1}}^{\theta_{k}}=\frac{x_{\Sigma_{1}}^{\theta_{k}}+\alpha \Sigma_{1}}{\left|x_{\Sigma_{1}}^{\theta_{k}}+\alpha \Sigma_{1}\right|}$. Using ${\hat{x}}_{\Sigma_{o1}}^{\theta_{k}}=\frac{x_{\Sigma_{o1}}^{\theta_{k}}+\alpha \Sigma_{o1}}{\left|x_{\Sigma_{o1}}^{\theta_{k}}+\alpha \Sigma_{o1}\right|}$ to relocate another half of $x_{\Sigma_{o1}}^{\theta_{k}}$, near to Σ*_o_*_1_. The density of relocated vectors is controlled by *α* (The illustration of *α* is given hereinafter). (5) Conduct local mean estimation based on conventional MEMD algorithm [22], while employing adaptive direction vectors $x_{\Sigma_{1}}^{\theta_{k}}$ and $x_{\Sigma_{o1}}^{\theta_{k}}$.

**Table 2:** The detailed procedures of adopting filter bank property of APIT-MEMD.

(1) Generate a set of same length Gaussian white noise (*m* channels) (2) Add the *m* channels Gaussian white noise in Step 1 of APIT-MEMD to the input *n*-channels signal, thus (*m* + *n*)-channels signal is obtained. (3) Conducting APIT-MEMD on the resulting (*m* + *n*)-variate signal to generate (*m* + *n*)-vairate IMF groups. (4) Discarding the *m*-variate IMF groups from the resulting (*m* + *n*)-vairate IMG groups, the obtained *n*-variate IMFs can be obtained.

**Table 3:** Characteristic frequencies of FAFNIR deep groove rolling bearing.

Fault Type	Fault Frequency (/Hz)
Inner ring fault	*f_i_* = 81.45
Outer ring fault	*f_o_* = 53.58

**Table 4:** Fault correlation factor of each IMF.

$\frac{\mathrm{IMF}\;\mathrm{order}}{\mathrm{FCF}}$	**1**	**2**	**3**	**4**	**5**
	0.1032	0.0974	0.1033	0.3525	0.32007
	6	7	8	9	10
	0.1210	0.0583	0.0652	0.0296	0.0067
